# Deletion of the Fission Yeast Homologue of Human Insulinase Reveals a TORC1-Dependent Pathway Mediating Resistance to Proteotoxic Stress

**DOI:** 10.1371/journal.pone.0067705

**Published:** 2013-06-24

**Authors:** Clémentine Beuzelin, Irini Evnouchidou, Pascal Rigolet, Anne Cauvet-Burgevin, Pierre-Marie Girard, Delphine Dardalhon, Slobodan Culina, Abdelaziz Gdoura, Peter van Endert, Stefania Francesconi

**Affiliations:** 1 Institut Curie, Centre de Recherche, Orsay, France; 2 Centre Nationale de la Recherche Scientifique, Unité Mixte de Recherche 3348, Centre Universitaire, Orsay, France; 3 Université Paris-sud XI, Orsay, France; 4 Université Paris Descartes, Sorbonne Paris Cité, Faculté de Médecine, Paris, France; 5 Institut National de la Santé et de la Recherche Médicale, Unité 1013, Paris, France; Iowa State University, United States of America

## Abstract

Insulin Degrading Enzyme (IDE) is a protease conserved through evolution with a role in diabetes and Alzheimer's disease. The reason underlying its ubiquitous expression including cells lacking identified IDE substrates remains unknown. Here we show that the fission yeast IDE homologue (Iph1) modulates cellular sensitivity to endoplasmic reticulum (ER) stress in a manner dependent on TORC1 (Target of Rapamycin Complex 1). Reduced sensitivity to tunicamycin was associated with a smaller number of cells undergoing apoptosis. Wild type levels of tunicamycin sensitivity were restored in *iph1* null cells when the TORC1 complex was inhibited by rapamycin or by heat inactivation of the Tor2 kinase. Although Iph1 cleaved hallmark IDE substrates including insulin efficiently, its role in the ER stress response was independent of its catalytic activity since expression of inactive Iph1 restored normal sensitivity. Importantly, wild type as well as inactive human IDE complemented gene-invalidated yeast cells when expressed at the genomic locus under the control of *iph1^+^* promoter. These results suggest that IDE has a previously unknown function unrelated to substrate cleavage, which links sensitivity to ER stress to a pro-survival role of the TORC1 pathway.

## Introduction

Human Insulin Degrading Enzyme (hIDE) or insulinase belongs to the M16A family of peptidases, which comprises large zinc-dependent metalloproteases found in all prokaryotic and eukaryotic organisms examined [1]. hIDE cleaves mainly small substrates, notably insulin for which it has high affinity, amyloid-beta, insulin-like growth factor II, transforming growth factor-α and monomeric ubiquitin [2], [3], [4]. Converging lines of evidence implicate hIDE in the pathogenesis of type 2 diabetes and of Alzheimer's disease [5], [6], [7]. However, its ubiquitous expression and evolutionary conservation support the notion of a conserved and broader role of IDE in cellular physiology. In this context, it has been proposed that IDE might specialize in degrading substrates prone to form amyloids, the accumulation of which is linked to proteotoxic stress [8]. However, a non-catalytic “dead-end chaperone” function of IDE has been proposed since it can form a highly stable complex with monomeric amyloid-beta, thereby preventing the formation of aggregates [9], [10]. In support of a role as a chaperone, hIDE is upregulated following different stresses with kinetics similar to those of heat shock proteins [11]. In addition, hIDE is the cellular receptor of Varicella-Zoster virus through binding but not clearance of virus glycoprotein E [12] and IDE with non-catalytic function has been found bound to androgen receptor in nuclear fraction of rat prostate cells [13].

The budding yeast orthologue of hIDE, Ste23p, displays similar substrate specificity as mammalian IDE and, together with Axl1, the second yeast M16A metalloprotease, cleaves the precursor of the mating pheromone a-factor [14], [15], that is expressed only in haploid cells. Indeed, cells lacking a functional Axl1 or Ste23 are sterile. Although processing of a-factor is the only known function of Ste23, it has been reported that, in contrast to Ax11, Ste23 is also expressed in diploid cells that do not produce pheromone, suggesting a function not yet determined not related to a-factor processing [14]. Thus, as for hIDE and insulin, Ste23 expression is not limited to the cell type expressing identified substrates.

Cellular proteins are subjected to continuous damage and maintenance of protein homeostasis is central to all biological processes. A cellular compartment particularly susceptible to protein damage is the endoplasmatic reticulum (ER). Accumulation of misfolded proteins in the ER induces the Unfolded Protein Response (UPR) that increases the level of chaperones, stimulates retro-translocation of misfolded proteins to the cytosolic proteolytic system and attenuates general translation and transcription. If this response cannot resolve the ER stress, apoptotic pathways are engaged [16], [17]. Insulin-producing pancreatic beta cells are a cell type particularly dependent on a functional UPR. In these cells, the UPR is constitutively activated to cope with glucose-triggered peaks of proinsulin production. Interestingly, we have found that IDE, the prototypical substrate of which is evidently insulin, is highly expressed in beta cells (PvE, unpublished), suggesting a possibly link between insulin degradation by IDE and control of the UPR in these cells.

The generation and resolution of cellular stress is intimately linked to the evolutionary conserved target of rapamycin (TOR) kinase, which regulates cell growth according to nutrient and energy availability [18]. Mammalian and yeast cells express two TOR complexes: TORC1 and TORC2. Higher eukaryotes have one TOR-encoding gene while fission yeast *Schizosaccharomyces pombe* has two: the non-essential *tor1^+^* gene encodes the kinase forming the TORC2 complex, while the essential *tor2^+^* gene encodes the kinase present in the TORC1 complex [19]. However, fission yeast Tor1 protein can function as part of the rapamycin sensitive complex TORC1 to control mitotic onset in response to nutrient stress [20]. On the contrary, Tor1 requirement for response to other stresses is not affected by rapamycin, indicating that under these conditions Tor1 functions in the TORC2 rapamycin insensitive complex [21]. Although rapamycin does not inhibit cell growth in fission yeast, it has been shown that it inhibits the Tor2 kinase activity towards the ribosomal S6 protein and that inhibition is reversed in the *tor2-S1837E* mutant, which is predicted to prevent interaction with the FKBP12-rapamycin complex [22]. More recent findings showed that fission yeast TORC1 activity is inhibited by FKBP12-rapamycin complex *in vitro*, similarly to other organisms [23].

Unbridled activation of mammalian TOR exacerbates cellular stress and is linked to diabetes, cancer and a shorter life span [24], [25]. Conversely, both yeast and mammalian TOR are required for the response to various types of stress including hypoxia, oxidative stress, DNA damage and proteotoxic stress [18], [26].

Several facts concerning IDE – its preference for amyloidogenic substrates able to cause cellular stress, its high expression in beta cells subjected to permanent ER stress, its dead-end chaperone function, indirect evidence for unidentified functions that might underlie its ubiquitous expression – prompted us to speculate that IDE might be implicated in the response to proteotoxic stress. To address this hypothesis, we took advantage of its evolutionary conservation and used the *S. pombe* homologue of mammalian IDE as model.

## Materials and Methods

### Strains and media

Strains are listed in Table 1. EMM medium was: 27.14 g/L of EMM powder without nitrogen (MP Biomedicals, Santa Ana, CA, USA) +5 g L-1 of NH4Cl. YNB medium was: 6.7 g/L of YNB W/Ammonium Sulfate (MP Biomedicals, Santa Ana, CA, USA). Both media were supplemented with 125 mg/mL of Arginine, Histidine, Uracile and Adenine and glucose at the final concentration of 2%. Yeast cultures were grown at 30°C otherwise stated. A Spodoptera frugiperda (Sf9) insect cell line was obtained from ATCC (Manassas, VA). Hi5 insect cells were described previsouly [27].

**Table 1:** Yeast strains used in this study.

*wt*	h- 972	lab's stock
*iph1-d*	h- iph1::kanR	this study
*iph1-d ura4+*	h- iph1::ura4+ ura4-D18 ade6-M216	this study
*iph1-M*	h- iph1::ura4+ ura4-D18 leu1-32 ade6-M210	this study
*tor1-d*	h-tor1::kanR	S. Moreno
*iph1-d tor1-d*	h- iph1::kanR tor1::ura4+ ura4-D18	this study
*tor2-51*	h+ tor2-51:ura4+ ura4-D18	S. Moreno
*iph1-d tor2-51*	h- iph1::kanR tor2-51:ura4+ ura4-D18	this study
*iph1-E71D*	h- iph1-E71D	this study
*hIDE*	h+ iph1::hIDE	this study
*hIDE-E111D*	h- iph1::hIDE-E111D	this study

### Gene disruption and replacement

Gene disruption and replacement were performed by PCR-based gene targeting [28]. Primers used to disrupt *iph1* with *KanR* marker: (5′TGGCCTCTAAACAGTAATGCCTACGTACTGTGTGTATGTAAACACATAATTCAACCTATTGCCATATTTCTTACATATTACGGATCCCCGGGTTAATTAA-3′) and (5′CTAGCAGAAGAGTAGGTCTCGTCACACTTGTTTGGATAGCGAGAAAAACCGCAGTGCCAGAATGCAAAACTGAAATTAAGGAATTCGAGCTCGTTTAAAC3′).

Primers used to to disrupt *iph1* with *ura4+* marker:

(5′TATTACCCTTTTTTTGGGTGTAATAGCAGTAGTCAGAATTCTGGGTTGTTTTATCTTTTCCTTTCATAAATAAAAACGCCAGGGTTTTCCCAGTCACGAC-3′) and (5′GTGCCAGAATGCAAAACTGAAATTAAGATGAGAATATAAAATCAGTAAATTTGAGAATCGGATTAGGGAAAAAAAAAGCGGATAACAATTTCACACAGGA).

Primers used to disrupt *tor1* with *ura4+* marker in strain *iph1-d ura4-D18*: (5′TGGAAGAATTGAACACCGCGACTATTAGAAAGTCTATCGTTTCACTCGCTCTCTTTGATTCATGGAGTATTTTAGTCGCCAGGGTTTTCCCAGTCACGAC 3′) and (5′TAAATTAATAACAACACGAAAAAAATTATCATAATCTCAAAAAACAGAAAACATCATTACCAAAAACTACACCATCAGCGGATAACAATTTCACACAGGA3′).

Primers used to replace the *iph1* coding sequence between position 188 and position 280 by the *ura4+* gene to obtain strain *iph1-M*:

(5′GAGAGATCCGGAAACAGATAATGCAAGTGCAGCTATTGACGTTCACATCGGCAGTCAAAGCAATCCACGAGAGTTGCGCCAGGGTTTTCCCAGTCACGAC 3′) and (5′ATAGAGCATCATGAGACACTTCGAAGTAATAATTTGTATTATTAGAGGCTGTATAGGCGTTTGAAATTCCATTATGAGCGGATAACAATTTCACACAGGA 3′).

Mutant *iph1-E71D* was obtained by replacing the *ura4+* gene in strain *iph1-M* with a DNA fragment of *iph1* gene mutated at codon 71 (GAA to GAT). This fragment was synthesized, cloned and sequenced by GeneArt (Regensburg, Germany).

The cDNA coding the hIDE protein was cloned into pCRBlunt plasmid. Site direct mutagenesis was used to change nt A390 to C to obtain the hIDE-E111D mutant as described in [29]. hIDE and hIDE-E111D coding regions were amplified using the following primers:

(5′ATTCAACCTATTGCCATATTTCTTACATATTACCCTTTTTTTGGGTGTAATAGCAGTAGTCAGAATTCTGGGTTGTTTTATCTTTTCCTTTCATAAATAAAAAATGCGGTACCGGCTAGCG 3′) and (5′TGTTTGGATAGCGAGAAAAACCGCAGTGCCAGAATGCAAAACTGAAATTAAGATGAGAATATAAAATCAGTAAATTTGAGAATCGGATTAGGGAAAAAAAACTAGAGTTTTGCAGCCATGAAGTTAATATG-3′).

PCR- fragments were used to transform the *iph1-d ura+* strain and transformants were selected on standard 5 fluoroorotic acid (5-FOA) medium. Stable transformants expressing hIDE and hIDE-E111D from the *iph1* promoter were selected.

### Iph1 alignment and modeling

Alignment was performed with Clustalw software. The tridimensional structure of Iph1 was performed using the Modeller software [30].

### Recombinant Iph1 expression and purification

A full length cDNA of 2911 bp encoding Iph1 was amplified using a high fidelity enzyme (Phusion, New England Biolabs, Evry, France) and primers encoding a C-terminal extension by six histidine residues, inserted into pCRBlunt (Invitrogen, Saint Aubin, France), sequenced completely to confirm the absence of errors, and transferred as XbaI/PstI fragment into the baculovirus transfer vector pVL1393 (Invitrogen). To produce the E71D mutant, site-directed mutagenesis was performed directly on the pVL1393-Iph1 plasmid. The primers used for the mutagenesis PCR were (5′GGATTGGCGCACTTTTGTGATCATCTGTTGTTTATGGGGAC3′) and (5′GTCCCCATAAACAACAGATGATCACAAAAGTGCGCCAATCC3′). After the PCR, the reaction mixture was digested with Dpn I and this material was used for transformation. Successful mutagenesis was confirmed by sequencing. Recombinant baculoviruses encoding wt Iph1 and Iph1-E71D were produced by co-transfection of Sf9 insect cells with the resulting plasmids and BaculoGold™ virus DNA, followed by a plaque assay and plaque selection by PCR. Recombinant Iph1 and Iph1-E71D were produced in Hi5 insect cells after infection with the recombinant baculoviruses. Cells were harvested 72 h post-infection and lysed on ice for 30 min in 25 mM Tris, 50 mM phosphate, 300 mM NaCl, 10 mM imidazole, 1% Triton X-100, pH 8.0 in the presence of protease inhibitors. The lysis supernatant was harvested by centrifugation for 10 min at 14000 rpm. Then it was transferred on Ni-NTA beads (Invitrogen) equilibrated in 50 mM phosphate, 300 mM NaCl, 10 mM imidazole, pH 8.0 and left to bind on a turning wheel at 4°C overnight. The resin was washed with the same buffer containing 10 mM imidazole, then with 25 mM imidazole and the protein was eluted with buffer containing 300 mM imidazole. The recombinant protein was used directly for enzymatic assays. Insect cell-expressed recombinant hIDE carrying an N-terminal extension by 7 His residues was purchased from R&D Systems (Lille, France).

### Measurement of enzymatic activity using a fluorogenic subsrate

Enzymatic activity towards the fluorogenic substrate Mca-RPPGFSAFK(Dnp) (Enzo Life Sciences, Villeurbanne, France) was measured by following the time-dependent increase in the fluorescence signal at 405 nm after excitation at 340 nm on a Mithras LB 940 plate reader (Berthold Technologies, Thoiry, France). 10 µM of substrate were incubated with 10 ng of hIDE or 20 ng of Iph1 in 50 mM Tris, 150 mM NaCl pH 7.4 at 25°C and fluorescence was recorded for 10 min. The resulting time slope was used to calculate the digestion rate of the fluorogenic substrate.

### Insulin digestions, analysis by RP-HPLC and SDS-PAGE

10 µg of insulin were incubated with 2–500 ng hIDE or 8–800 ng Iph1 in 50 mM Tris, 150 mM NaCl pH 7.4 for 3 h 20 min or 16 h in 300 µl final volume. Reactions were stopped by the addition of 30 µl 10% formic acid. Analysis of insulin digestions was performed by reversed phase HPLC on a μRPC C2/C18 ST 4.6/100 column (GE Healthcare, Velizy-Villacoublay, France). Digestion products were eluted using a 20–50% acetonitrile gradient, while monitoring the absorbance at 215 nm. For analysis by SDS-PAGE on 15% Tris-Tricine gels, 185 ng insulin were digested for 16 h with Iph1 WT, hIDE or Iph1 E71D (0.7, 3.5 or 14 ng). Bands were visualized using SYPRO Orange Protein Gel Stain (Sigma-Aldrich, Lyon, France).

### Proteotoxic stress

In drop tests, cells from exponentially growing cultures in either EMM or YNB were treated or not with tunicamycin (TU; Sigma, St. Louis, MO, USA) for 45 min, serially diluted in H_2_O and spotted onto the indicated medium. TU, dithiotreitol (DTT; Sigma) and rapamycin (RA; Sigma) treatments were performed on mid-log cultures grown in YNB. For each time point or drug concentration two to three dilutions were plated on YNB in triplicates and plates incubated at the appropriated temperature for 3–5 days. Colonies formed were counted and percent of survival calculated against time 0. All experiments shown were performed at least three times. TU was suspended in dimethyl sulfoxide (DMSO) at 10 mg/mL and used at 10 µg/mL otherwise stated. RA was suspended in DMSO at 0.5 mg/mL, and used at the final concentration of 300 ng/mL. DTT was suspended in H_2_O at 1 M and used at the final concentration of 50 mM.

### Western blot

Protein extracts were prepared as described in [31]. Proteins were resolved in 4–12% bis-Tris gels and transferred onto nitrocellulose. Proteins were detected using antibodies specific for IDE (clone 9B12; a generous gift of R.A. Roth, Stanford Univ.), phospho-Akt substrate (including p27; Cell Signaling, Danvers, MA, USA) and Hog1 (Santa Cruz Biotechnology, Santa Cruz, CA, USA) as loading control. Quantification of bands was done using ImageJ software.

### Metacaspase activation

Cells were treated with 10 µg/ml of TU for 45 min, collected, suspended in fresh medium without TU and incubated at 30°C with agitation. At 0, 4 and 6 hours from release, cells were processed as described in [32] to assess metacaspase activation using the fluorescent probe FITC-VAD-FMK (CasPACE, Promega, WI, USA). Fluorescence was detected with a FACSCalibur flow cytometer (Becton Dickinson, NJ, USA) as described in [32] and data analyzed using CellQuest software.

## Results

### The*S. pombe* genome encodes a homologue of hIDE

**Figure 1:**
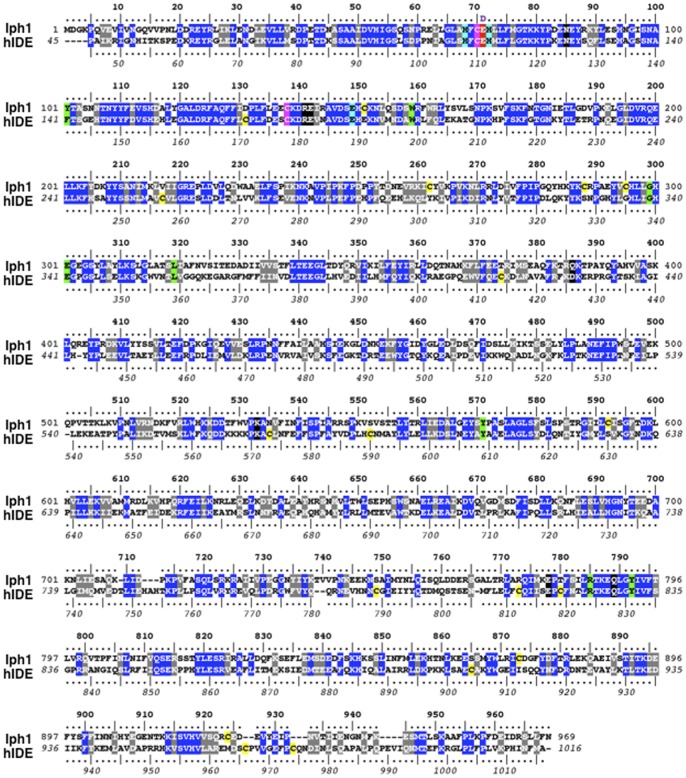
Sequence alignment of*S.* *pombe* Iph1 and hIDE. Identities are box-shaded in blue and similarities in grey. Non-conserved Cys residues are shaded in yellow and conserved Cys residues in magenta. Residues important for catalysis are box-shaded as follows: residues required for Zn^2+^ coordination, cyan; Glu residue involved in catalysis, red (E71 in Iph1 and E111 in hIDE); E71/E111 were substituted by Asp in strains *iph1-E71D* and *hIDE-E111D*. Residues important for substrate recognition and fixation are shaded in green and those required for interaction between the catalytic and the substrate binding domains are shaded in black [48].

The genome of *S. pombe* encodes five putative metallopeptidases belonging to the M16 peptidase family that are potential orthologues of budding yeast Ste23p. Among these, the SPACUNK4.12c ORF on chromosome 1 encodes a protein sharing the highest degree of identity (37%) with hIDE (Fig. 1). As shown in Table 2, the degrees of identity between hIDE and the other putative metallopeptidases of *S. pombe* belonging to the M16 peptidase family range between 8% and 13.5%. We will refer to the *S. pombe* SPACUNK4.12c ORF as *iph1^+^* gene for *i*nsulinase *p*ombe *h*omologue 1.

**Table 2:** Sequence comparisons of hIDE and some peptidases of the M16 family.

Metallopeptidase (*gene*)	Protein accession number	Family-Subfamily	Organism	% Identity with hIDE	Length (Nb residues)
insulysin	Q9JHR7	M16-A	*Mus musculus*	94.9%	1019
insulysin	P22817	M16	*D. melanogaster*	45.4%	990
insulysin	NP_507226	M16-A	*C. elegans*	37.6%	985
Iph1 (*SPACUNK4.12c*)	O14077	M16-A	*S. pombe*	37%	969
insulysin	AEE79660	M16-A	*A. thaliana*	32.6%	881
Uncharacterized protein (*SPAC3H1.02c*)	Q10068	M16-C	*S. pombe*	13.5%	1036
Cym1 (*SPBC119.17*)	O42908	M16-C	*S. pombe*	13.2%	882
Qcr1 (*SPBP23A10.15c*)	Q9P7X1	M16	*S. pombe*	10.2%	457
Mas2 (*SPBC18E5.12c*)	O94745	M16-B	*S. pombe*	10%	502
Qcr2 (*SPCC613.10*)	P78761	M16-B	*S. pombe*	8.1%	426

Fission yeast Iph1 protein, predicted to be cytosolic, lacks the first 40 amino acids reported to direct a fraction of hIDE to mitochondria [33]. Sequence alignment of Iph1 with hIDE shows that residues required for substrate recognition, catalysis and interaction between the N- and the C-terminal domains are conserved (Fig. 1), supporting the notion that Iph1 is an orthologue of hIDE.

**Figure 2:**
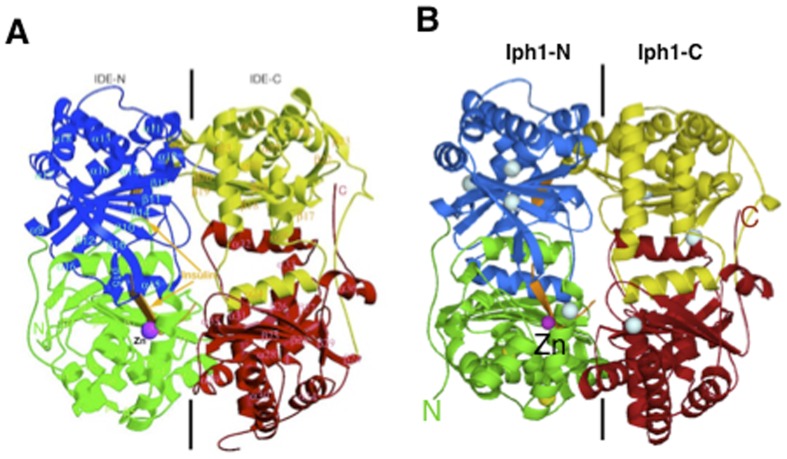
Structures of hIDE and model of Iph1 in complex with the insulin B chain. *A*) Crystallographic structure of the human IDE-E111Q–insulin B chain complex (PDB code 2G56) [48]. Domains 1, 2, 3 and 4 are colored green, blue, yellow and red, respectively. The Zn^2+^ ion and insulin B chain are colored magenta and orange, respectively. *B*) Model of an insulin B chain/Iph1 complex. The four domains of Iph1 are drawn in the same orientation and color codes as in *A*. The two Cys conserved in hIDE are in yellow, the remaining Cys residues are in cyan.

The high degree of similarity between Iph1 and hIDE allowed computing a tridimensional structure of the *S. pombe* protein using the crystallographic structure of hIDE as a template (Fig. 2).

### Iph1 and hIDE have similar protease activity

**Figure 3:**
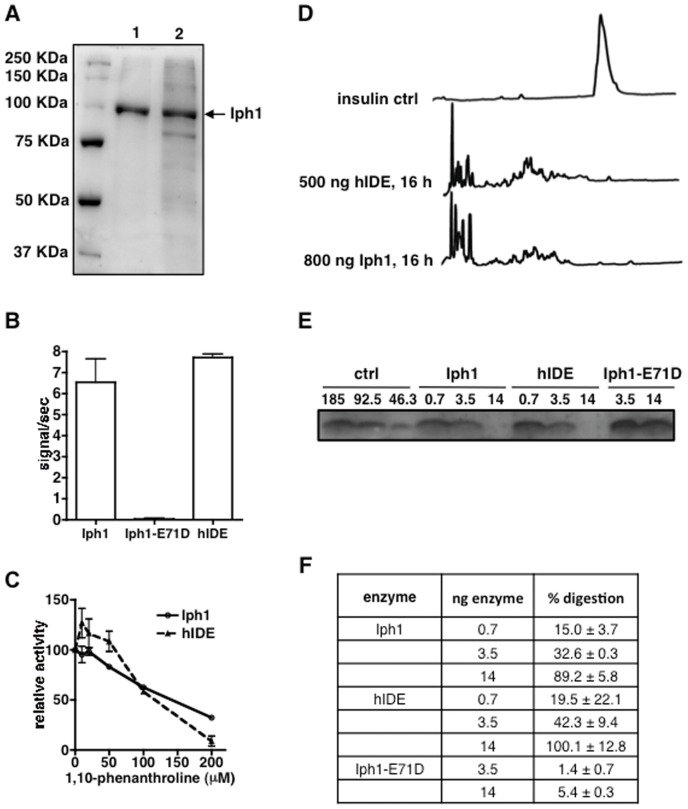
Iph1 and hIDE have similar protease activity. *A*) SDS-PAGE of Iph1 elution fractions at 300 mM imidazole after Ni-NTA purification. 1: Iph1 wt, 2: Iph1-E71D. *B*) Activity of Iph1 WT, Iph1-E71D and hIDE towards Mca-RPPGFSAFK(Dnp). Error bars represent the standard deviation of 3 experiments. *C*) Titration of 1,10-phenanthroline inhibition of Mca-RPPGFSAFK(Dnp) digestion by Iph1 or hIDE. Activity is expressed as the percentage of activity of the enzyme in the absence of inhibitor. The error bars represent the standard deviation of duplicates. *D*) Reversed phase HPLC analysis of insulin digestions. Insulin was digested for 16 h at 37°C in the presence of 500 ng hIDE or 800 ng Iph1. *E*) Tricine SDS-PAGE analysis of insulin digestions. 185 ng insulin were digested for 16 h with Iph1 wt, hIDE or Iph1-E71D (0.7 or 3.5 or 14 ng). The left hand lanes show different dilutions of undigested recombinant insulin, corresponding to 100%, 50% and 25% of the starting amount used in digestions. One out of 3 experiments performed is shown. *F*) The percentage of digestion by the different enzymes was calculated by measuring insulin band intensity on Tricine SDS-PAGE gels.

In order to compare the protease activity of Iph1 with hIDE we expressed recombinant wt Iph1 and a mutant Iph1 protein with a substitution of residue Glu71 in the catalytic site by Asp, both tagged by six C-terminal His residues, using the baculovirus system. The equivalent mutation in hIDE (E111D) decreases enzyme activity to<1% without affecting substrate binding [34]. The purified proteins were>99% pure and migrated at the expected molecular weight as viewed by SDS-PAGE (Fig. 3A). We measured protease activity towards the fluorogenic IDE-specific substrate Mca-RPPGFSAFK(Dnp). Comparison with digestion by hIDE shows that the human and yeast wt enzymes have similar protease activity whereas the E71D mutant has no measurable proteolytic activity (Fig. 3B). In addition, titration of the digestion reaction with increasing concentrations of 1, 10-phenanthroline, a metallopeptidase inhibitor, shows that the two enzymes are inhibited to an almost identical extent (Fig. 3C). Addition of increasing concentrations of Zn^2+^ to the fluorogenic peptide digestion had an equivalent inhibitory effect for both enzymes (data not shown).

Next, we sought to determine whether Iph1 is able to cleave insulin, the hallmark natural substrate of hIDE cleaved with high efficiency by it. We performed insulin digestions with various amounts of enzyme and analyzed the results by column and gel chromatography. After 16 h of digestion with either 500 ng hIDE or 800 ng Iph1, the insulin peak was no longer detectable in reversed phase chromatography and various new product peaks appeared on the chromatogram (Fig. 3D). Analysis of insulin digestions by Tricine SDS-PAGE confirmed gradual loss of intensity of the band corresponding to insulin in the presence of increasing concentrations of the two enzymes, whereas no significant degradation was seen for the Iph1-E71D mutant (Fig. 3E). We quantified band intensities to determine the percentage of insulin digestion by the enzymes and observed that the Iph1 and hIDE are almost equally capable of digesting insulin (Fig. 3F). In conclusion, in addition to their high degree of identity and conservation of residues critical for catalysis, Iph1 and hIDE display strikingly similar protease activity and specificity, consistent with functional conservation.

### *iph1-d* protects cells from proteotoxic stress

To study Iph1 function we constructed a haploid strain where the *iph1* ORF was replaced by the selectable marker *KanR*. Cells disrupted for *iph1* (*iph1-d*) did not show any obvious phenotype and, in contrast to budding yeast *ste23* null mutant, cells were not sterile.

IDE is highly expressed in murine pancreatic beta cells (our unpublished observation), in which the UPR is constitutively activated to handle glucose-triggered bursts of insulin synthesis that challenge the ER protein folding capacity [35], [36]. Speculating that IDE might therefore be implicated in dealing with ER stress, we compared the survival to TU of cells lacking Iph1 (*iph1-d*) to wild-type (*wt*) cells. TU is an ER stressor that blocks protein N-glycosylation in the ER, leading to accumulation of misfolded proteins.

**Figure 4:**
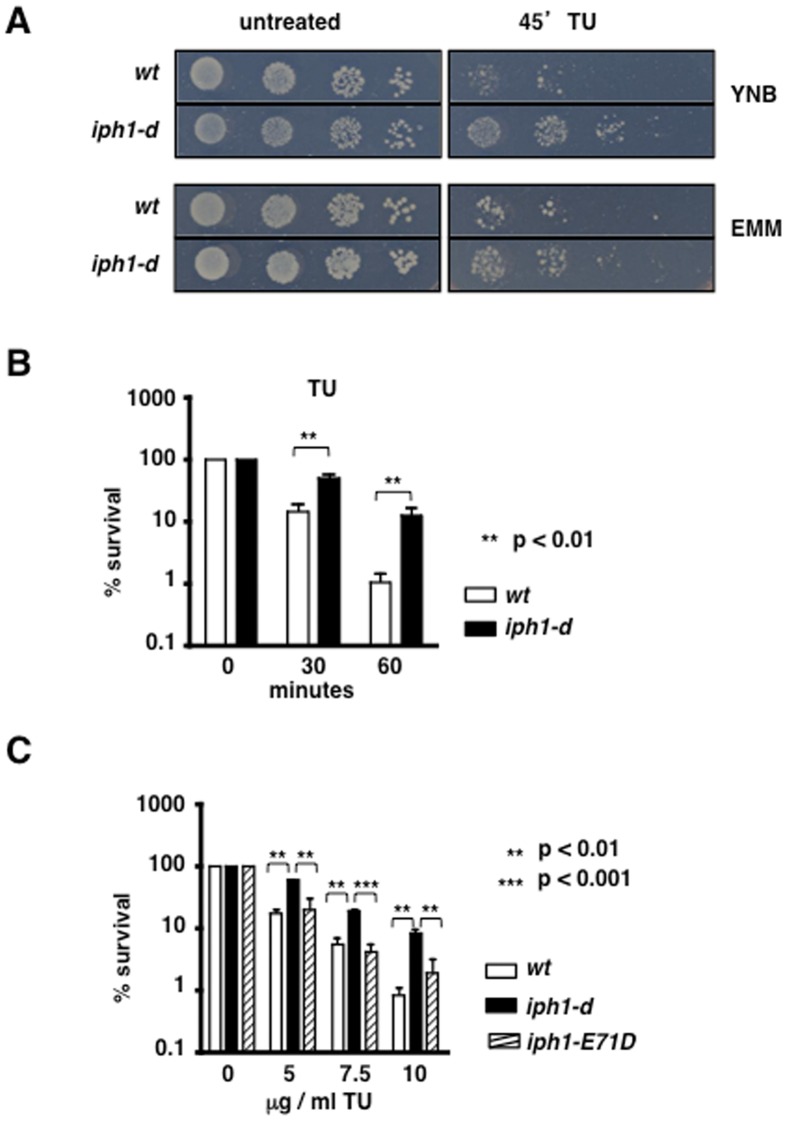
Lack of Iph1 protects cells from ER stress. *A*) Drop test of *wt* and *iph1-d* strains grown in the indicated medium and exposed or not to TU for 45 min. *B*) Survival of the indicated strains to TU. The mean (+/− S.E.M) of seven independent experiments is shown. Mann Whitney test was used for statistical analysis. *C*) Survival to 1 h treatment with different concentrations of TU of *wt*, *iph1-d* and protease mutant *iph1-E71D*. For all strains the mean (+/− S.E.M.) of three independent experiments is shown. ANOVA with Tukey's test was applied for statistical analysis.

We found that after 45 min of treatment with TU, *iph1-d* cells were more resistant to induced ER stress than *wt* (Fig. 4A) when grown in EMM or YNB, two synthetic minimal media. We confirmed this result by measuring survival of cultures grown in YNB and exposed to 10 µg/ml TU for 0, 30 and 60 min. After 60 min of treatment *wt* survival was around 1% while *iph1-d* survival was 12% (Fig. 4B).

We noticed that strains prototroph for leucine were more sensitive to ER stress than auxotroph strains. Therefore all experiments shown were performed with prototroph strains cultivated in minimal YNB medium.

Given that proteins belonging to the insulinase family have protease activity, we asked if increased survival to ER stress of *iph1-d* cells was due to lack of Iph1 protease function. Thus, a strain expressing only the Iph1 protein mutated at the residue E 71 in the catalytic site (*iph1-E71D*) was constructed by replacing in the haploid strain the *iph1^+^* sequence with *iph1-E71D*. As shown in Fig. 3, this mutant is catalytically inactive. As shown in Fig. 4C, expression of inactive Iph1 reconstituted normal TU sensitivity at all tested concentrations, indicating that the phenotype of *iph1-d* cells is due to lack of the protein but not to lack of its protease activity. Thus, Iph1 has a function in the ER stress response that is unrelated to substrate degradation.

#### Tor1 is not required for resistance of*iph1-d* cells to TU and DTT

**Figure 5:**
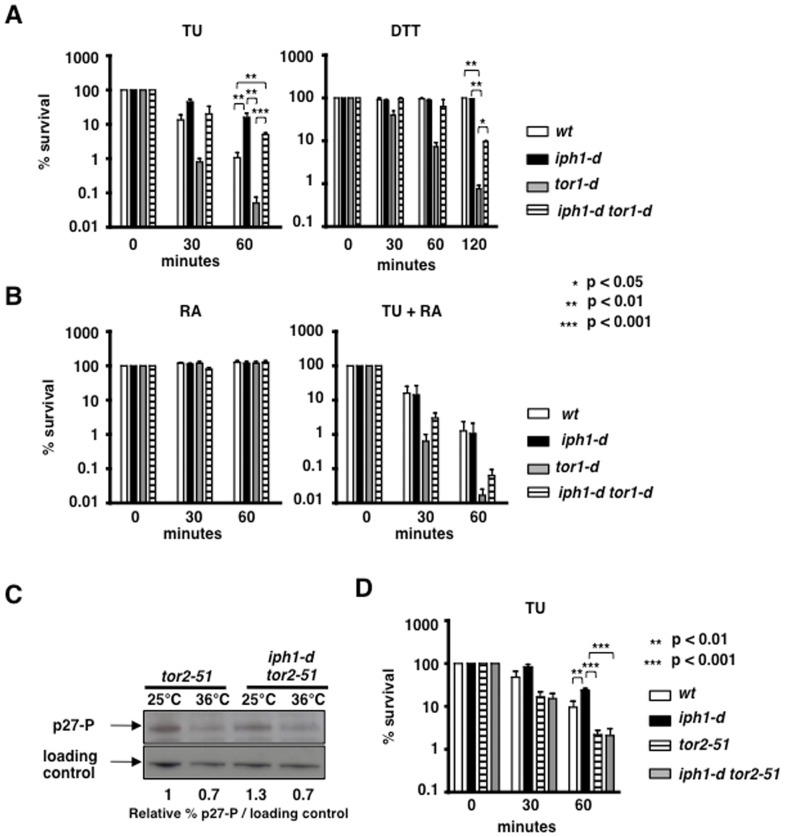
Iph1 loss suppresses the sensitivity to proteotoxic stress of*tor1* mutant in a TORC1-dependent manner. *A*) Survival to TU (left panel) and DTT (right panel) of the indicated strains. For all strains the mean (+/− S.E.M.) of four independent experiments is shown. *B*) Survival to RA or TU and RA of the indicated strains. For all strains the mean (+/− S.E.M.) of three independent experiments is shown. *C*) Cell extracts from *tor2-51* and *iph1-d tor2-51* cells grown at 25°C and shifted to 36°C for 45 min were probed with anti-phospho-AKT Substrate antibodies recognizing the phosphorylated form of p27. Sty1 was detected as loading control, using anti-Hog1 antibodies. *D*) Survival to TU of the indicated strains. The mean (+/− S.E.M.) of four independent experiments is shown. For all experiments ANOVA with Tukey's test was applied for statistical analysis.

Fission yeast Tor1 kinase is required for the response to a wide range of stresses including heat stress and DNA damaging conditions [21], [37], [38]. We found that cells deleted for *tor1* (*tor1-d*) were slightly more sensitive than *wt* cells to TU in all experiments, however differences in survival between *wt* and *tor1-d* were at the limit of statistical significance (*p* = 0.05) (Fig. 5A, right panel). In contrast, *tor1-d* cells were significantly more sensitive to DTT, a proteotoxic thiol reducing agent (Fig. 5A, right panel). The higher sensitivity to DTT of *tor1-d* cells is likely due to wider effects of DTT on the cellular environment in contrast to specific inhibition of *N*-acetylglucosamine transferases by TU. However, double mutant *iph1-d tor1-d* cells survived TU treatment to the same extent as *iph1-d* cells (Fig. 5A left panel), indicating that deletion of the *iph1* gene rendered *tor1-d* cells more resistant to TU. Deletion of *iph1* also partially suppressed the DTT sensitivity of *tor1-d* cells (Fig. 5A right panel). Thus, resistance to proteotoxic stress conferred by lack of Iph1 is independent of the Tor1 pathway. On the contrary, deletion of *iph1* did not suppress the sensitivity to heat [37], [38] or to genotoxins [21] of *tor1-d* cells (not shown), indicating that the observed phenotype might be specific to ER stress.

### TU resistance of*iph1-d* cells is abolished by rapamycin and by heat inactivation of Tor2

Next we asked whether suppression of the TU sensitivity of *wt* and *tor1-d* cells by *iph1* deletion might depend on the Tor2 kinase that participates in the formation of the RA sensitive TORC1 complex as shown in [22], [23], [39]. Treatment for 1 hour with rapamycin (RA) alone did not affect survival of any strain (Fig. 5B left panel). RA treatment also did not modify the sensitivity to TU of *wt* cells and of *tor1-d* cells (Fig. 5A and B). In contrast, concomitant treatment with TU and RA abrogated the TU resistance conferred to *wt* and *tor1-d* cells by *iph1* deletion (compare panel B right to panel A left in Fig. 5). Thus, resistance to TU conferred by *iph1-d* to *wt* and *tor1-d* relies on a RA sensitive function, likely the Tor2 protein kinase. Because RA does not affect the TU response in *wt* cells, we can speculate that a RA sensitive factor, likely the TORC1 complex, is not required for the normal response to ER stress. However, in the absence of the Iph1 protein, this RA sensitive function acquires the capacity to protect cells from ER stress.

We took advantage of the thermosensitive allele *tor2-51* to corroborate this conclusion. This allele has no phenotype at the permissive temperature of 25°C while protein activity is rapidly lost in a reversible way at 36°C, the non-permissive temperature [40]. Cells were grown at 25°C, shifted to 36°C for 45 min prior to addition of TU to half of each culture. Cells were plated to measure survival at 0, 30 and 60 min of treatment and incubated at the permissive temperature of 25°C. To study the efficacy of this protocol, we monitored the Tor2 kinase activity towards its known substrate p27 at 25°C and after 45 min of incubation at 36°C, using published protocols [22]. p27 phosphorylation was reduced after incubation at 36°C in both *tor2-51* and *iph1-d tor2-51* cells, confirming heat inactivation of Tor2 kinase activity (Fig. 5C). Short incubation at 36°C in the absence of TU did not affect the survival of any strain (not shown) and *tor2-51* cells were as sensitive to TU than *wt*, while *iph1-d* cells were significantly more resistant to TU. In line with the experiments with RA, the double mutant *iph1-d tor2-51* was as sensitive as *tor2-51* and as *wt* cells to TU (Fig. 5D), confirming that temperature inactivation of Tor2 prevents TU resistance of *iph1-d* cells. Note that growing cells at 25°C instead of 30°C reduced TU sensitivity.

**Figure 6:**
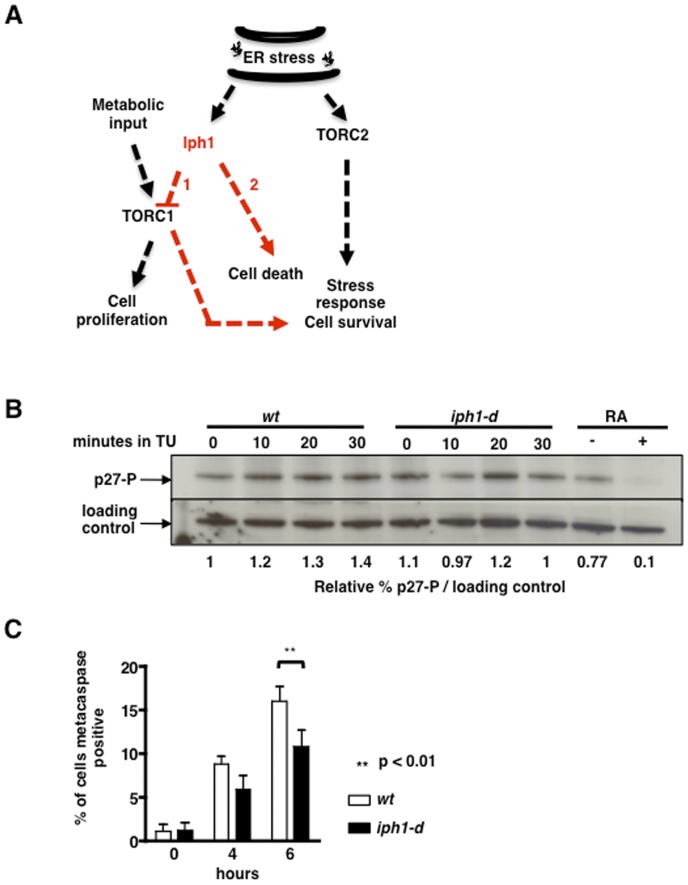
Iph1 has a pro-apoptotic function during ER-stress. *A*) Model resuming the principal findings in this study. See text for explanation. *B*) Cell extracts from *wt* and *iph1-d* cells exposed to TU for the indicated times and cell extracts from *wt* cells exposed or not to RA were probed with antibodies recognizing the Tor2-dependent phosphorylated form of p27. Sty1 was detected as loading control, using anti-Hog1 antibodies. *C*) *wt* and *iph1-d* cells were treated or not with TU for 45 min and released in fresh medium for 0, 4 and 6 hours. At each time point the metacaspase activity was detected with the fluorescent probe FITC-VAD-FMK. Bars are the mean of three independent experiments. At 6 hours the percentage of metacaspase positive test is statistically different with a *p* value of 0.0046.

Thus, resistance to ER stress of *iph1-d* cells is abolished by RA treatment and by heat inactivation of Tor2. Therefore TORC1 complex activity is required for protection from ER stress in *iph1-d* cells. One possibility is that TORC1 activity is upregulated in *iph1-d* cells and that this accounts for resistance to ER stress. This scenario could be encountered if Iph1, presumably after activation by ER stress, acts as a direct or indirect inhibitor of TORC1 (scenario 1 in Fig. 6A). Thus, we monitored Tor2 kinase activity towards p27 in *wt* and *iph1-d* cells exposed to TU for different times. As a control *wt* cells were exposed to RA that fully inhibits Tor2 kinase activity. As shown in Fig. 6B, no clear upregulation of Tor2 activity towards the p27 substrate was observed in mutant cells exposed to TU.

An alternative scenario would be that Iph1 might physiologically direct cells to apoptosis upon sustained ER stress, bypassing a TORC1-dependent rescue pathway (scenario 2 in Fig. 6A). Indeed, it is well established that UPR activation in ER stressed cells will eventually result in apoptotic death if ER homeostasis is not re-established [16], [17]. To test this possibility, we measured metacaspase activation in *wt* and *iph1-d* cells exposed to TU. Cultures in log phase were treated with TU for 45 min, collected and released in fresh medium without the drug. At different time points an aliquot of cells was processed with the fluorescent marker FITC-VAD-FMK to monitor metacaspase activation. Quantification of metacaspase positive cells in two independent experiments indicated that around 16% of *wt* cells were undergoing apoptosis 6 hours after release from the treatment. In contrast, around 10% of *iph1-d* cells contained active metacaspase (Fig. 6C). This suggests that resistance to ER stress in *iph1-d* cells correlates with a lower number of cells engaged in the apoptotic pathway.

### hIDE complements*iph1-d*

**Figure 7:**
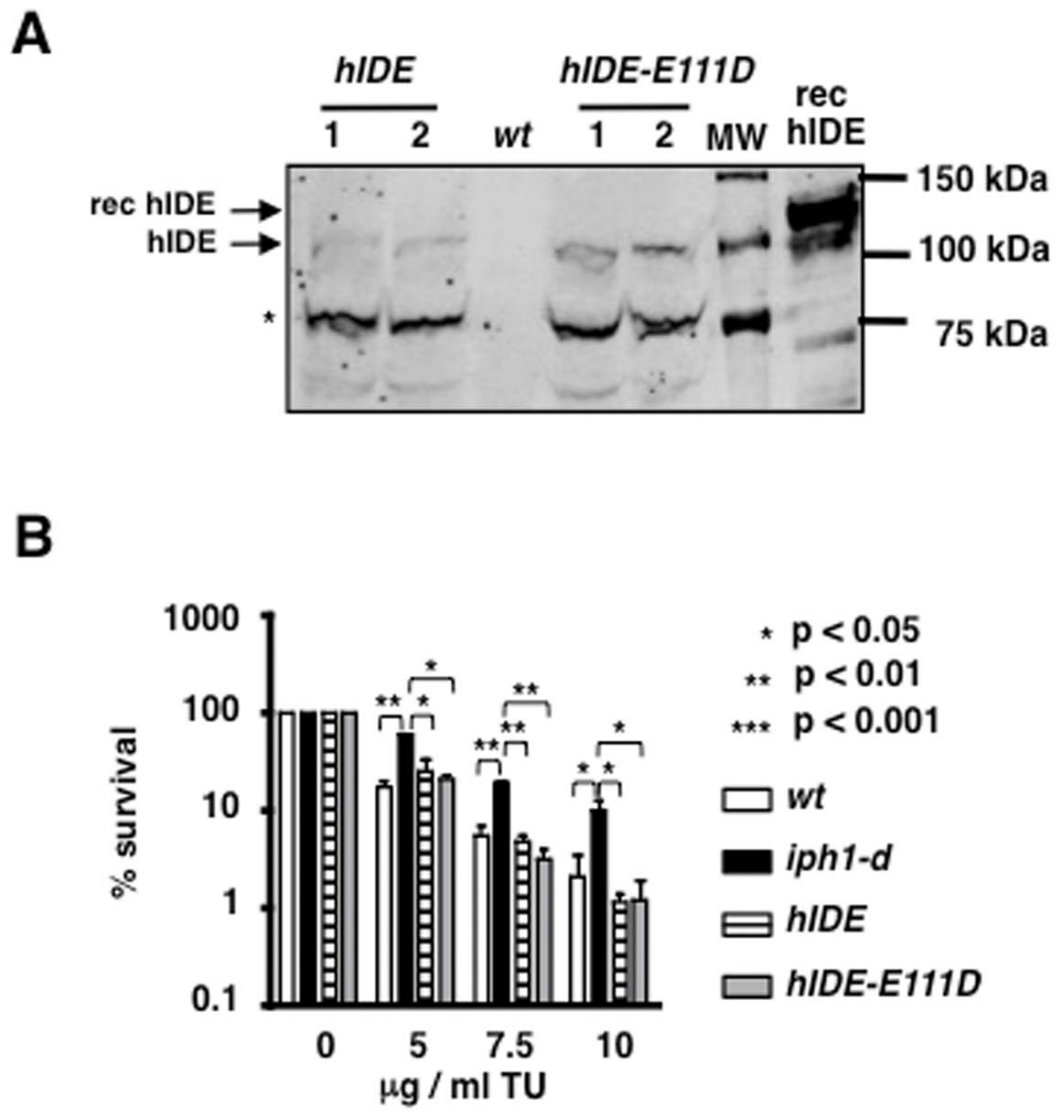
hIDE and hIDE-E111D complement*iph1-d* cells when expressed at the genomic locus. *A*) Cell extracts from two independent clones (1 and 2) expressing hIDE or hIDE-E111D at the *iph1* genomic locus were probed with anti-IDE antibodies. Wt indicates protein extract from a strain not expressing the human protein. rec hIDE indicates purified recombinant hIDE carrying a poly-His extension with an expected MW of 114 kDa. The star indicates a possible degradation product. *B*) Survival to 1 h treatment with different concentrations of TU of the indicated strains. The mean (+/− S.E.M.) of three independent experiments is shown and ANOVA with Tukey's test was applied for statistical analysis.

Our results reveal a function of the fission yeast homologue of hIDE that is independent of its protease activity. Wondering whether this function might be conserved during evolution, we asked if hIDE could replace the fission yeast homologue and if the protease function of hIDE was required for this. Therefore we constructed strains containing a single copy of the *hIDE* or *hIDE-E111D* cDNAs under the control of the *iph1* promoter and at the *iph1* genomic locus. Protein expression was confirmed by immunoblot (Fig. 7A) [41]. Survival to TU of these strains was monitored after 60 min of incubation with different concentrations of drug. As shown in Fig. 7B, cells expressing hIDE or hIDE-E111D displayed a similar sensitivity to TU than *wt* cells. Thus, hIDE as well as the mutant hIDE-E111D can substitute for the *S. pombe* Iph1 protein indicating that human IDE can exert a protease-independent function in yeast cells experiencing ER stress.

## Discussion

In this work we have identified the fission yeast IDE homologue, named Iph1, and first shown that its protease activity is remarkably similar to that of its human counterpart hIDE, consistent with the high degree of sequence conservation between the two proteins, and with a model suggesting similar structures. However, the principal finding of this study is that Iph1 has an additional function unrelated to substrate cleavage that modulates the physiological response to ER stress, which also appears to be conserved between Iph1 and hIDE. This finding might account for the expression of insulinase in a wide range of eukaryotic cells and tissues which, as for fission yeast, lack known substrates.

The protection from ER stress in cells lacking Iph1 is abolished upon functional silencing of the TORC1 complex by RA treatment or by heat inactivation of Tor2 protein, linking Iph1 to the TOR pathway. While these experiments suggest that TORC1 activity renders *iph1-d* cells more resistant to ER stress, we have shown that the levels of p27 phosphorylation, known to be a TORC1 substrate in *S. pombe*, are not increased in *iph1-d* cells, arguing against an hyper-activation of TORC1 in the absence of Iph1 (scenario 1 in Fig. 6A). However, it cannot be ruled out that p27 is not a substrate of TORC1 in *iph1-d* cells under conditions of ER stress. According to an alternative scenario (2 in Fig. 6A) during ER stress, Iph1 might physiologically activate a pathway leading ultimately to cell death that bypasses and/or overrides a TORC1-dependent survival pathway, which therefore would be unveiled only in its absence. In this case, upregulation of TORC1 in *iph1-d* cells would not necessarily be expected. Measurement of metacaspase activation in TU treated cells showed lower numbers of metacaspase positive cells in the *iph1-d* strain compared to *wt*, supporting the notion that Iph1 might be involved in engaging cells towards apoptosis upon ER stress thus explaining the increased survival of *iph1-d* cells.

At first sight, our finding of a pro-survival effect of TORC1 in the presence of ER stress is in conflict with some published findings. Thus, upregulation of mTORC1 in cells lacking the tuberous sclerosis complex, an inhibitor of TOR activity, induces ER stress and the UPR [24] and mTORC1 activation has a pro-apoptotic effect in cells experiencing ER stress [42]. However, in our experiments inhibition of the TORC1 pathway by rapamycin did not change the sensitivity to TU of *wt* cells, demonstrating that in yeast cells and under our experimental conditions, TORC1 does not promote apoptosis in cells experiencing ER stress. We propose that this apparent discrepancy may be due to different baseline levels of TORC1 activity in the different systems. In our study, TORC1-dependent protection from ER stress was observed in cells grown in synthetic but not in rich medium. Although synthetic medium does not differ from rich medium with respect to the concentration of glucose, the main carbon source for *S.pombe*, it supports a lower growth rate. Cellular growth is determined by a complex and incompletely understood interplay between gene expression, cell cycle regulation, stress response and energy metabolism, in which TORC1 plays a central role, adjusting the growth rate to environmental conditions. It is plausible to assume that TORC1 activation was low in our system while it was high or normal in the studies suggesting a pro-apoptotic effect of TORC1 during ER stress. Therefore our data suggest that the effect of TORC1 on cell survival and apoptosis may differ according to its activation level, with a pro-survival effect at low to normal activation contrasting with a pro-apoptotic effect at supra-normal levels. Note that mTORC1 also plays a dual role in ER stress response, with a pro-survival function during acute ER stress that stimulates the pro-survival kinase Akt upstream of mTORC1, and an anti-survival function during chronic ER stress due to inhibition of Akt by mTORC1 itself [43]. Thus, Iph1 might be required in *wt* cells to balance TORC1 outputs in response to ER stress.

Next to activating the UPR, ER stress is an inducer of autophagy that plays a cytoprotective role by preventing accumulation of misfolded proteins in apoptosis proficient cells [44]. However, several considerations argue against the hypothesis that Iph1 deficiency could protect from ER stress by increasing autophagy. First given that Tor2 inactivation (but not RA) induces autophagy in *S. pombe* [45], [46], this hypothesis would predict that heat inactivation of Tor2-51 should further increase resistance to ER stress rather than reduce it as experimentally observed. Moreover, reduced TORC1 activation and upregulation of autophagy is known to extend the lifespan and delay aging [47], however *iph1-d* cells do not display delayed aging (our unpublished observation).

We have shown that hIDE shares the protease independent function of Iph1 at least in yeast cells. Our results suggest that knock-out of mammalian IDE might also lead to ER stress resistance at least under certain metabolic conditions. This might be particularly relevant for pancreatic beta cells, in which excessive ER stress is linked to functional failure and diabetes development. The known capacity of IDE to degrade insulin is commonly viewed as mechanistic underpinning of its genetic link to diabetes. Our results suggest considering an additional or alternative possibility, namely that an implication of IDE in the response to proteotoxic stress might affect the survival of pancreatic beta cells in this pathology.
